# Supplementary material for: Predicting the tensile properties of heat treated and non-heat treated LPBFed AlSi10Mg alloy using machine learning regression algorithms
Source: PLoS One. 2025 Jun 2;20(6):e0324049. doi: 10.1371/journal.pone.0324049 (PMC12129343; doi:10.1371/journal.pone.0324049)
Supplement: Attachment — Submitted filename: Response to the Comments - PONE-D-24-46880R1.docx [file pone.0324049.s003.docx]

***Authors’ Response to Reviewers Comments***

| ***Manuscript ID*** | PONE-D-24-46880R1 |
| --- | --- |
| ***Manuscript Title*** | Predicting the Tensile Properties of Heat Treated and Non-Heat Treated LPBFed AlSi10Mg Alloy using Machine Learning Regression Algorithms |
| ***Authors*** | Vijaykumar S Jatti, Saiyathibrahim A, Murali Krishnan R, Vinaykumar S Jatti, Ashwini V Jatti, Savita V Jatti |

**The authors would like to thank the Editor and Reviewers for their valuable time to review the manuscript. The manuscript has been revised carefully based on the comments, and the details are as follows.**

**Responses to Comments**

All the modifications are highlighted in the revised manuscript.

**Reviewer#1 comments**

***Comment 1:***

The quality of the pictures in this article is generally poor, please upload high-quality pictures and add some picture labels.

***Authors’ Response:***

- The suggested comment is accepted.
- As per the suggestion, all the figures are enhanced in high quality and also separately submitted with revised manuscript for further proceedings.
- Kindly see page numbers 7, 13, 14, 16, 17, 18, 19, 20, 26, 29 and 32.

***Comment 2:***

The second chapter of the article has more conceptual content, please simplify the conceptual content and supplement the experimental data content involved in this article.

***Authors’Response:***

- The suggested comment is accepted.
- As per the advice, the content of entire chapter 2. Materials and Methods has been simplified and some experimental details are added in the revised manuscript.
- Kindly see page numbers 9, 11, 12, 15, 20 and 21.

***Comment 3:***

Although RMSE, MSE, MAE and R^2^ are used to evaluate the model, more statistical analysis methods can be added in the discussion to support the superiority statement of the model.

***Authors’ Response:***

- The suggested comment is accepted.
- More statistical analysis methods such as Mean Bias Error (MBE), Mean Absolute Percentage Error (MAPE) and Adjusted R^2^ were included.
- Kindly see page numbers 22, 23, 24, 25, 27, 28, 30 and 31.

***Comment 4:***

If the model has been hyper-parameterized, it can be clearly stated in the paper, which can help readers understand whether the reported performance indicators are the best results for each model.

***Authors’ Response:***

- The suggested comment is accepted.
- The Gaussian Process Regression (GPR) model using Bayesian Optimization/Grid Search, where key hyperparameters were tuned to maximize performance. The following hyperparameters were adjusted:

**Kernel Function:** Radial Basis Function (RBF) Kernel with automatic relevance determination

**Length Scale:** Tuned between [0.1, 10]

**Noise Level (σ²):** Optimized within [10⁻³, 1]

**Optimizer:** Limited-memory BFGS (L-BFGS) algorithm

**Cross-validation:** 5-fold cross-validation

This tuning process ensured that the reported performance metrics represent the best possible results for the model.

- The following details are included in the revised manuscript. Kindly see page numbers 12 and 13.

***Comment 5:***

The training process of the prediction model is less, such as: optimizer, activation function, neuron, number of iterations, learning rate and other data

***Authors’ Response:***

- The suggested comment is accepted.
- The training process details for various models are included in the revised manuscript.
- Kindly see page numbers 12, 13, 14, 15, 20 and 21.

***Comment 6:***

The paper mentions data augmentation, ' Data was augmented up to a limit of 100 Iterations... ', why there is no overfitting in the number of iterations within 100 times, please explain this part in more detail.

***Authors’ Response:***

- The suggested comment is accepted.
- Explanation related to data augmentation and overfitting prevention has been included in the revised manuscript.
- Kindly see page number 9.

***Comment 7:***

Although the effect of heat treatment on tensile properties (such as tensile strength and yield strength) was reported, the results showed inconsistencies, which may be confusing to readers. It is recommended to explain the reasons for these inconsistencies, such as changes in the heat treatment process, material batch differences, or factors such as the size / geometry of the sample. It is necessary to further explain why heat treatment does not generally improve the ductility and other properties, which will contribute to the practical application value of the research.

***Authors’ Response:***

- The suggested comment is accepted.
- The reasons for the inconsistencies, such as changes in the heat treatment process, material batch differences, or factors such as the size/geometry of the sample, are also reasons for heat treatment not generally improving the ductility and other properties that are also discussed in the revised manuscript.
- Kindly see page number 22 and 23.

***Comment 8:***

Although the conclusions summarize the research results well, the practical application implications of these results can be further discussed, such as: how the model can help guide the design of LPBF aluminum alloy components, and whether these findings can affect the decision-making in the manufacturing process.

***Authors’ Response:***

- The suggested comment is accepted.
- As per the advice, the practical application and suitability of the model can guide during the design of LPBF aluminum alloy components, and the effect of the study’s findings on the decision-making in the manufacturing process is discussed in the conclusion section.
- Kindly see page number 34.

**Reviewer#2 comments**

***Comment 1:***

Carefully review the text to identify and correct minor typographical errors, such as the one in the sentence following Equation 2.

***Authors’ Response:***

- The suggested comment is accepted.
- Minor typographical error in the manuscript and sentence following Equation 2 was rectified.
- Kindly see page number 8.

***Comment 2:***

Enhance the quality and resolution of the figures, particularly Figures 4 and 5, to improve clarity.

***Authors’Response:***

- The suggested comment is accepted.
- As per the advice, the quality and resolution of the figures 4 and 5 are improved. Kindly see page numbers 16, 17 and 18 of the revised manuscript.

***Comment 3:***

Improve the discussion on the tensile test results, emphasizing the differences between treated and untreated samples. Use new references to facilitate this discussion.

***Authors’ Response:***

- The suggested comment is accepted.
- The discussion on the tensile test results, emphasizing the differences between heat treated and non heat treated samples are included in the revised manuscript. Kindly see page numbers 22 and 23.
- Further, new citations have been included for this discussion. Kindly see references 44 and 45 at page numbers 39 & 40.

***Comment 4:***

The conclusions are too long, it is necessary to summarize them.

***Authors’ Response:***

- The suggested comment is accepted.
- As per the suggestion, the conclusion section is summarized in the revised manuscript.
- Kindly see page numbers 33 and 34.

Authors are thankful to the Editors and Reviewers.
